# Phylogenetic Position of the Genus *Alulacris* (Orthoptera: Acrididae: Melanoplinae: Podismini) Revealed by Complete Mitogenome Evidence

**DOI:** 10.3390/insects12100918

**Published:** 2021-10-08

**Authors:** Haiyang Xu, Benyong Mao, Sergey Yu. Storozhenko, Yuan Huang, Zhilin Chen, Jianhua Huang

**Affiliations:** 1 Key Laboratory of Cultivation and Protection for Non–Wood Forest Trees (Central South University of Forestry and Technology), Ministry of Education, Changsha 410004, China; haiy_xu@126.com; 2 Guangxi Key Laboratory of Rare and Endangered Animal Ecology, Guangxi Normal University, Guilin 541004, China; 3 Key Laboratory of Forest Bio-Resources and Integrated Pest Management for Higher Education in Hunan Province, Central South University of Forestry and Technology, Changsha 410004, China; 4 College of Agriculture and Biology Science, Dali University, Dali 671003, China; maoby65@sohu.com; 5 Federal Scientific Center of the East Asia Terrestrial Biodiversity, Far East Branch of the Russian Academy of Sciences, 690022 Vladivostok, Russia; storozhenko@biosoil.ru; 6 College of Life Sciences, Shaanxi Normal University, Xi’an 710119, China; yuanh@snnu.edu.cn

**Keywords:** *Alulacris* *shilinensis*, Podismini, Melanoplinae, mitogenome, phylogenetic position

## Abstract

**Simple Summary:**

The phylogenetic position of the genus *Alulacris* is clarified based on complete mitogenome evidence. The results show that *Alulacris* consistently has the closest relationship to the genus *Yunnanacris* of the subfamily Melanoplinae in all phylogenetic trees and is extremely similar to *Yunnanacris* *yunnaneus* (Ramme, 1939) morphologically. Therefore, the genus *Alulacris* is transferred here from Catantopinae *incertae* *sedis* to the nominal subtribe Podismina of the tribe Podismini *sensu* Ito (2015) of the subfamily Melanoplinae.

**Abstract:**

Whole mitogenomes are a useful data source for a wide variety of research goals due to the vastly cheaper sequencing cost and the far less demanding high-quality templates. The mitogenome has demonstrated great potential in resolving phylogenetic questions in Orthoptera at different taxonomic scales as well as exploring patterns of molecular and morphological character evolutions. In this study, the complete mitogenome of *Alulacris* *shilinensis* (Zheng, 1977) was sequenced using next-generation sequencing, the characteristics of the mitogenome are presented briefly, and the phylogeny of the Melanoplinae and Catantopinae was reconstructed using a selected dataset of mitogenome sequences under maximum likelihood and Bayesian inference frameworks. The results show that the genus was consistently assigned to the subfamily Melanoplinae rather than Catantopinae in all phylogenetic trees deduced from different datasets under different frameworks, and this finding is entirely consistent with its morphological characters. Therefore, it is more appropriate to place the genus *Alulacris* in Melanoplinae rather than in Catantopinae.

## 1. Introduction

While the availability of other types of “–omics” data, in particular transcriptomes, is increasing rapidly, whole mitogenomes are still a useful data source for a wide variety of research goals due to the vastly cheaper sequencing cost and the far less demanding high-quality templates [1,2]. The mitogenome can be sequenced not only individually but also as a byproduct of all other ‘–omics’ approaches. The mitogenome has become the most widely used genomic resource available for systematic entomology over the past decade and now has been sequenced for all insect orders and in many instances representatives of each major lineage within orders (suborders, series or superfamilies depending on the group). These data have been applied to resolve systematic questions at all taxonomic scales from interordinal [3,4,5], through many intraordinal [6,7,8,9] and family-level relationships [10,11], to population/biogeographic studies [12].

As mentioned in a previous study [13], there are many controversial taxa in Acrididae, which have been designated different taxonomic positions by different authors. These issues must be resolved step by step as part of obtaining an improved classification scheme. The successful case study to clarify the phylogenetic positions of four genera in the subfamily Oxyinae using mitogenome data provides a valuable approach for resolving such issues [13].

The genus *Alulacris* is endemic to China, with *Pseudogerunda* *shilinensis* Zheng, 1977 as the type species and with three known species so far [14,15,16,17,18]. According to the original description, *Alulacris* is most similar to the genus *Circocephalus* Willemse, 1928 [15]. While not classified to any subfamily in the original reference, *Alulacris* was unambiguously considered a member of the subfamily Podisminae *sensu* Li & Xia [16], a taxonomic group corresponding to the tribe Podismini of the subfamily Melanoplinae [19]. However, in the Orthoptera Species File (OSF), it is placed without a definition of the tribe in the subfamily Catantopinae, possibly according to the general rule to treat the taxa of grasshoppers with a prosternal process [13,19]. Since the mitogenome has demonstrated great potential in resolving phylogenetic questions in Orthoptera at different taxonomic scales [12,13,20,21,22,23,24,25,26,27,28,29,30,31,32,33,34] as well as exploring patterns of molecular and morphological character evolutions [35,36,37,38], we assess in this study the phylogenetic relationship of the genus *Alulacris* with the candidates of its close relatives through a mitochondrial phylogenomics approach and clarify the current position of this genus in the modern classification of grasshoppers.

## 2. Materials and Methods

### 2.1. Taxon Sampling

The type species, i.e., *Alulacris* *shilinensis* (Zheng, 1977), was selected as the representative of the genus *Alulacris*. The material of *Alulacris* *shilinensis* for generating mitogenome data (voucher number: mt1811) was collected at Shilin Scenic Spot, Shilin County, Kunming city, Yunnan Province, China; 103°19′16″ E, 24°48′19″ N, altitude 1778 m; on 15 August 2017, Bing Jiang leg. The specimens were identified by the last author according to the keys to species in Li & Xia’s (2006) and the Mao et al. (2011) monographs [16,17]. They were preserved in anhydrous ethanol and are stored at room temperature in the Insect Collection of the Central South University of Forestry and Technology.

Considering the fact that Catantopinae used to be defined by a series of negatives as a dumping ground for all those genera, which cannot be placed into any of the other subfamilies with a prosternal process [28,39], we included in this analysis nearly all presently available mitogenome data of species in Acrididae and Dericorythidae with a prosternal process (Supplementary Materials Table S1), representing 12 subfamilies and 48 genera in total. Among these genera, *Choroedocus* was provisionally considered a member of the subfamily Eyprepocnemidinae due to its high similarity to the genera of Eyprepocnemidinae. *Coryphistes ruricola* (MG993389, MG993390, MG993403, MG993406) in Catantopinae and *Kosciuscola tristis* (MG993402, MG993408, MG993414) in Oxyinae were not included in the analysis because they have only partial mitogenome sequences available [28]. 

All material was collected under appropriate collection permits and approved ethics guidelines.

The morphological terminology followed that of Uvarov (1966) [40] and Storozhenko et al. (2015) [41]. The terminology of male genitalia followed that of Dirsh (1956) [42].

### 2.2. Sequencing, Assembly, and Annotation

A hind femur of male *Alulacris* *shilinensis* was sent to Novogene Biotech Co., Ltd. (Beijing) for genomic sequencing using next-generation sequencing (NGS), and the remainder of the specimen was deposited as a voucher specimen (voucher number: mt1811) at the Central South University of Forestry and Technology. Whole genomic DNA was extracted from muscle tissue of the hind femur using a modified routine phenol/chloroform method. Separate 400-bp insert libraries were created from the whole genome DNA and sequenced using the Illumina HiSeq X Ten sequencing platform. Twenty Gb of 150-bp paired-end (PE) reads were generated in total for each sample.

Raw reads were filtered to remove reads containing adaptor contamination (>15 bp matching the adaptor sequence), poly-Ns (>5 bp Ns), or >1% error rate (>10-bp bases with quality score <20). The mitogenome sequence was assembled from clean reads in Mitobim [43], and two runs were implemented independently using the same reference with different starting points (one point was *trnI* and the other was *COX1*) to improve the sequence quality of the control region. The assembled raw mitogenome sequences were primarily annotated online using the MITOS Web Server (http://mitos.bioinf.unileipzig.de/index.py; 18 May 2021) [44] and were then checked and corrected in Geneious R11 [45]. The secondary structure of the RNA encoding genes predicted in MITOS were visualized and checked manually using VARNA [46]. The newly sequenced mitogenome of *Alulacris* *shilinensis* has been deposited in GenBank under accession number MW810985 (Table 1). Base composition, A-T- and G-C-skews, and codon usage were calculated in MEGA X [47]. The formulas used to calculate the skews of the composition were (A − T)/(A + T) for the A−T-skew and (G − C)/(G + C) for the G−C-skew.

### 2.3. Phylogenetic Analyses

To clarify the phylogenetic position of *Alulacris* *shilinensis*, 94 complete mitogenome sequences representing 88 species in total, including 34 species in Melanoplinae and nine species in Catantopinae, were selected as ingroups, and four other Acridomorpha species, which included two species in Pamphagidae of Acridoidea and two in Pyrgomorphoidea, served as outgroups (Table S1). Three different datasets were prepared, which consisted of (1) the 13 protein-coding genes (PCGs), (2) the 2 rRNA genes, or (3) the combination of the 13 PCGs and the two rRNA genes.

The protein-coding genes were codon-based aligned using the MUSCLE algorithm in the TranslatorX online platform (http://translatorx.co.uk; 5 June 2021) [48] and toggled back to their nucleotide sequences, and the rDNA sequences were individually aligned in MUSCLE using default parameters. Finally, the alignments were manually optimized and concatenated into three different datasets using SequenceMatrix v.1.7.8 [49].

The dataset was divided into 41 data blocks (13 PCGs divided into individual codon positions and two ribosomal genes).The best-fitting models of nucleotide evolution and best-fitting partitioning schemes were selected using ModelFinder [50], and the models used for the phylogenetic analyses are shown in Table 1. The phylogenies were reconstructed in maximum likelihood (ML) and Bayesian inference (BI) frameworks. The ML phylogenies were reconstructed using IQ-TREE [51], and the approximately unbiased branch support values were calculated using UFBoot2 [52]; the analysis was performed in W-IQ-TREE [53] using the default settings. Nodes with a bootstrap percentage (BP) of at least 70% were considered well supported in the ML analyses [54]. BI analyses were accomplished in MrBayes 3.2.1 (http://morphbank.Ebc.uu.SE/mrbayes/; 10 June 2021) [55], with two independent runs, each with four Markov Chain Monte Carlo (MCMC) chains. The analysis was run for 1 × 10^7^ generations, sampling every 100 generations, and the first 25% generations were discarded as burn-in, whereas the remaining samples were used to summarize the Bayesian posterior probabilities (BPP). BPPs > 0.95 were interpreted as strongly supported [56].

## 3. Results

### 3.1. Characteristics of the Mitogenome of Alulacris Shilinensis

The complete mitogenome of *Alulacris* *shilinensis* is a circular molecule with a total length of 16950 bp (Table 2). It has the typical metazoan mitochondrial gene set consisting of 13 PCGs, 22 tRNAs, two rRNAs (the large and small ribosomal subunits), and a putative A + T-rich region (control region, CR). Among the 37 genes coded by the mitogenome, 23 genes are coded at the J strand and 14 at the N strand. The gene order is the same as that of other published mitogenomes in Caelifera and is similar to the ancestral type of gene arrangements in Ensifera [23], with the only difference in the order of *trnD* and *trnK*. The order of *trnD* and *trnK* between *COX2* and *ATP8* is *K*→*D* in Ensifera, but the order in Caelifera is *D*→*K*. The base composition is clearly A-T biased with a total A + T content of 74.1% (Table 2). The total A-T- and G-C-skews are 0.1120 and -0.1274, respectively.

All PCGs have a typical initiation codon of ATN, with eight PCGs (*ND2*, *COX2*, *ATP6*, *COX3*, *ND4*, *ND4L*, *ND6*, and *CYTB*) initiated from ATG, two from ATC, two from ATT, and one from ATA (Table 3). In terms of termination codons, the majority of PCGs have a typical termination codon of TAA, with *ND1* terminated by TAG, *ND2* by the incomplete termination codon TA, and *COX1* by T. The PCGs of the mitogenome have extremely similar codon usage pattern to other grasshoppers (Table 4). Among all codons of the PCGs, the most preferred codon with the highest average relative synonymous codon usage (RSCU) is UUA that codes for Leucine (Table 4) and has an RSCU value of 4.13%. The next common codons are UCA and UCU that code for Serine, followed by CGA (Glycine) and ACA (Threonine), with average RSCU values of 2.78%, 2.71%, 2.67% and 2.41%, respectively, indicating a distinct codon usage bias in grasshoppers [13].

The sizes of the 22 tRNAs vary over a very small range from the minimum of 64 bp for *trnP* to the maximum of 71bp for *trnK* and *trnV* (Table 3). Except for *tRNASer-AGN* lacking the DHU arm, all of the other 21 tRNAs can be folded into a typical clover structure (Supplementary Materials Figure S1).

The *lrRNA* and *srRNA* are located between the *trnL1-* and *trnV-* and the A + T-rich regions, respectively. Their lengths are 1377 bp (*lrRNA*) and 793 bp (*srRNA*), respectively. The control region is located between *rrnS* and *trnI*, with a length of 2121 bp, and, similar to most of the mitogenome, contains a high proportion of the A + T content of 72.5% (Table 2).

### 3.2. Phylogeny

**Figure 1:**
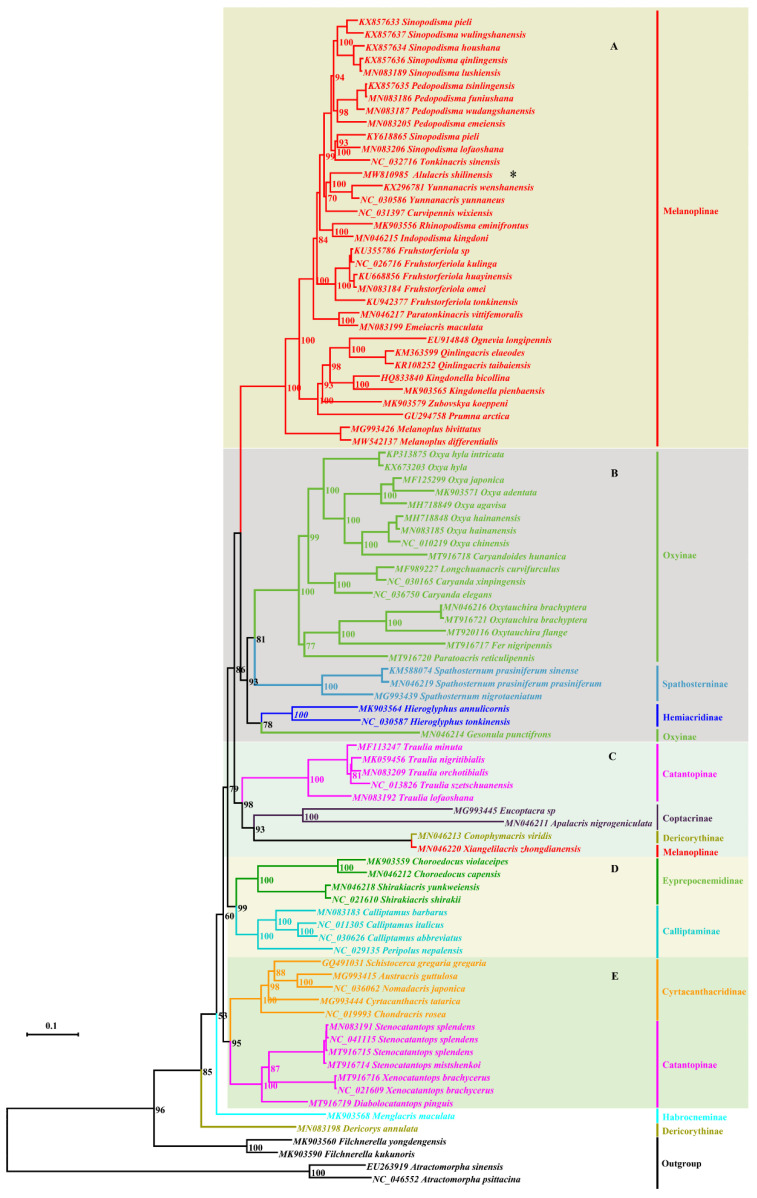
Phylogenetic tree reconstructed from the sequences of the 13 mitogenome PCGs (protein-coding genes) using maximum likelihood. The asterisk indicates the species *Alulacris* *shilinensis*. (**A**). Melanoplinae (excluding the genus *Xiangelilacris*). (**B**). Oxyinae + Spathosterninae + Hemiacridinae. (**C**). Coptacrinae + *Traulia* + *Conophymacris* + *Xiangelilacris*. (**D**). Eyprepocnemidinae+Calliptaminae. (**E**). Cyrtacanthacridinae+Catantopinae.

**Figure 2:**
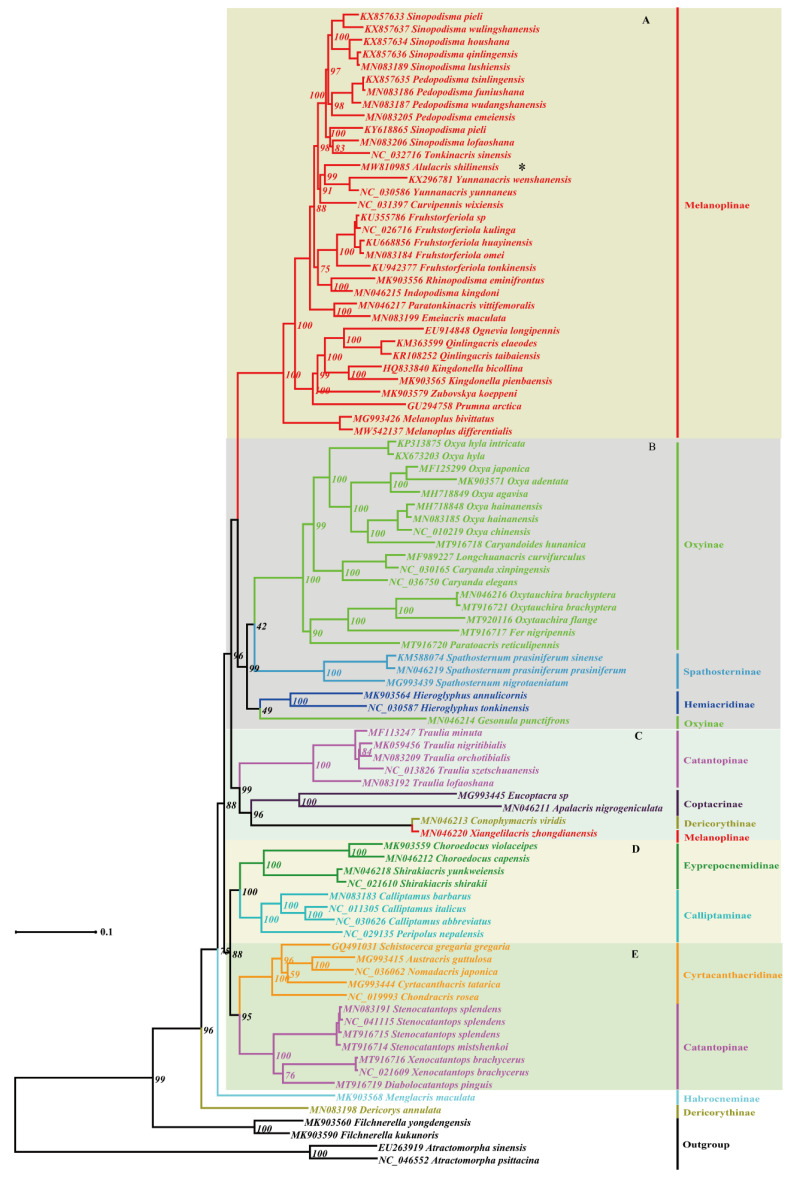
Phylogenetic tree reconstructed from sequences of the 13 mitogenome PCGs (protein-coding genes) and two rRNAs using maximum likelihood. The asterisk indicates the species *Alulacris* *shilinensis*. (**A**). Melanoplinae (excluding the genus *Xiangelilacris*). (**B**). Oxyinae + Spathosterninae + Hemiacridinae. (**C**). Coptacrinae + *Traulia* + *Conophymacris* + *Xiangelilacris*. (**D**). Eyprepocnemidinae + Calliptaminae. (**E**). Cyrtacanthacridinae+Catantopinae.

**Figure 3:**
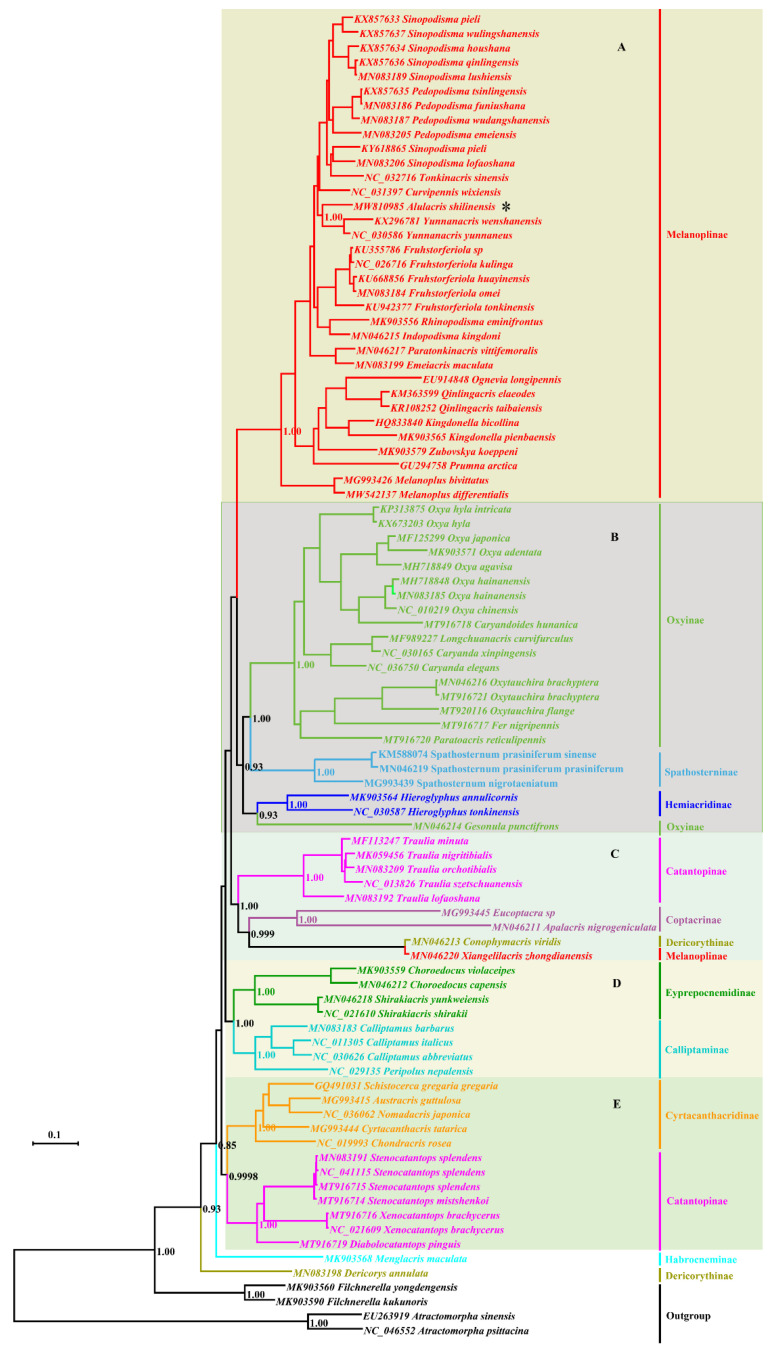
Phylogenetic tree reconstructed from sequences of the 13 mitogenome PCGs (protein-coding genes) using Bayesian inference. The asterisk indicates the species *Alulacris* *shilinensis*. (**A**). Melanoplinae (excluding the genus *Xiangelilacris*). (**B**). Oxyinae + Spathosterninae + Hemiacridinae. (**C**). Coptacrinae + *Traulia* + *Conophymacris* + *Xiangelilacris*. (**D**). Eyprepocnemidinae+Calliptaminae. (**E**). Cyrtacanthacridinae+Catantopinae.

**Figure 4:**
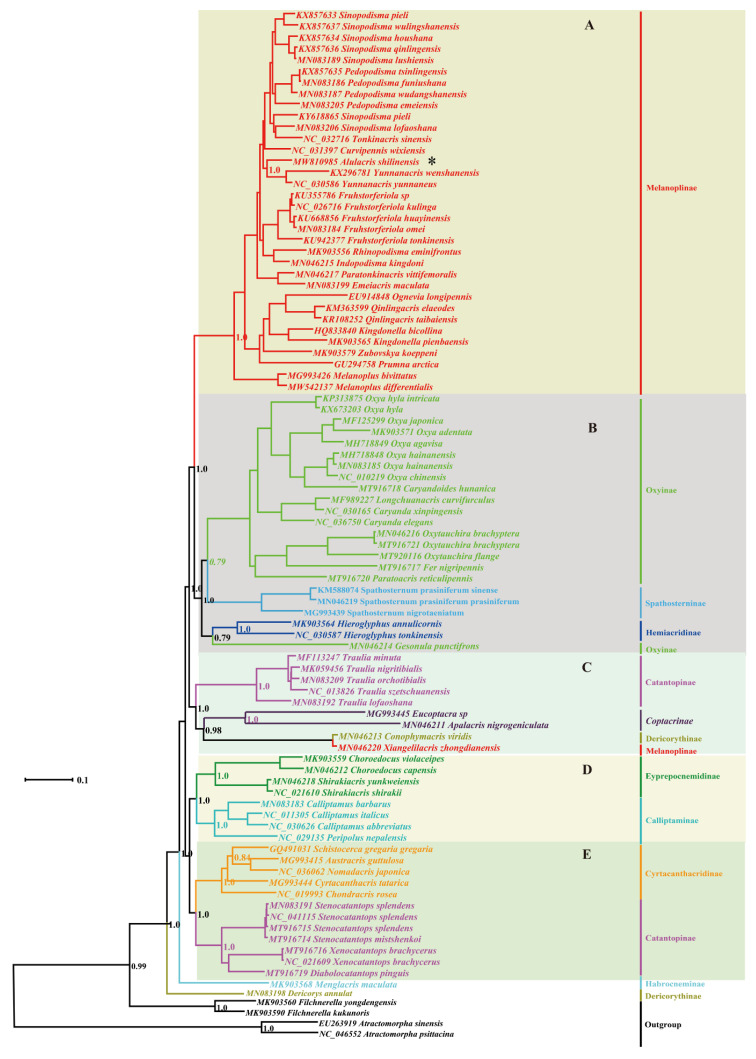
Phylogenetic tree reconstructed from sequences of the 13 mitogenome PCGs(protein-coding genes) and two rRNAs using Bayesian inference. The asterisk indicates the species *Alulacris* *shilinensis*. (**A**). Melanoplinae (excluding the genus *Xiangelilacris*). (**B**). Oxyinae + Spathosterninae + Hemiacridinae. (**C**). Coptacrinae + *Traulia* + *Conophymacris* + *Xiangelilacris*. (**D**). Eyprepocnemidinae+Calliptaminae. (**E**). Cyrtacanthacridinae+Catantopinae.

Maximum Likelihood and Bayesian Inference analyses produced largely congruent topologies from the PCGs and combined datasets, and five clades were consistently retrieved for the ingroup in all trees usually with high supporting values or posterior probabilities (Figure 1, Figure 2, Figure 3 and Figure 4): (A) Melanoplinae (excluding the genus *Xiangelilacris*), (B) Oxyinae+Spathosterninae+Hemiacridinae, (C) Coptacrinae+*Traulia*+*Conophymacris*+*Xiangelilacris*, (D) Eyprepocnemidinae+Calliptaminae, and (E) Cyrtacanthacridinae+Catantopinae. The only difference in the relationship between clades is the position of clade D, which clusters with clade E in the trees from PCGs+rRNA sequences (Figure 2 and Figure 4), and with Clade C in those from PCGs sequences (Figure 1 and Figure 3). Clade E is well supported with a bootstrap value of 95% in the ML trees (Figure 1 and Figure 3) and with a posterior probability of 1 in the BI trees (Figure 3 and Figure 4). The relationship within clades is also extremely robust.

The monophyly of eight groups (Spathosterninae, Hemiacridinae, Coptacrinae, Eyprepocnemidinae, Calliptaminae, Cyrtacanthacridinae, the Catantopini clade, and the genus *Traulia* of the subfamily Catantopinae) is always well supported, and the monophyly of Melanoplinae is supported except for *Xiangelilacris* *zhongdianensis,* which clusters into a separate clade with *Conophymacris* *viridis*. Similarly, the monophyly of Oxyinae is supported, except for one species, *Gesonula* *punctifrons,* which clusters first with species of the subfamily Hemiacridinae. 

At the species level, *Alulacris* *shilinensis* consistently has the closest relationship with the genus *Yunnanacris* (Melanoplinae, Podismini) in all trees, and the clade comprising the genera *Alulacris* and *Yunnanacris* is always extremely robust with a bootstrap value of 99–100% or a posterior probability of 1 and is located near the tip of the trees (Figure 1, Figure 2, Figure 3 and Figure 4).

Despite the high congruence among the trees from the PCG and PCG+rRNA sequences, the topology of the trees from the two rRNA genes is slightly different (Figures S2 and S3). Although three of the five clades retrieved in the trees from the PCG and PCG+rRNA sequences are still retrieved in the trees from rRNAs sequences, both clades C and E are split into two individual clades, the clade of Coptacrinae shows a closer relationship with the clade of Catantopini than with that of *Conophymacris*+*Xiangelilacris*; the placement of *Alulacris* *shilinensis*, *Gesonula* *punctifrons,* and *Menglacri* *smaculata* has little change, and the relationship among *Alulacris shilinensis*, *Curvipennis wixiensis,* and three other groups is unresolved (Figure S3). At a deeper level, the relationship among clades of Calliptaminae+Eyprepocnemidinae, Cyrtacanthacridinae, and the remaining groups is also unresolved (Figure S3). The relationship within clade B is a little different from that in the trees from the PCG and PCG+rRNA sequences, i.e., the subclade of Spathosterninae clustered first with *Gesonula punctifrons* and then with the subclade of Hemiacridinae, but not first with that of the majority of Oxyinae. 

## 4. Discussion

According to the modern classification of grasshoppers, the subfamily Catantopinae is the largest subfamily of Acrididae, with 326 genera and 998 species widely distributed in the Eastern Hemisphere, including Palaearctic and Oriental regions, Australia, and Africa [19,57,58,59]. Previously, all grasshopper species with the prosternal process were grouped into Catantopinae *sensu lato* [60], but now the definition has been reduced. Catantopinae is not defined by any distinct morphological feature, and was usually used as a dumping ground for all genera with a prosternal process which cannot be placed into any of the other subfamilies [39]. Moreover, this subfamily is a non-monophyletic group [28,61,62].

On the contrary, monophyly of the subfamily Melanoplinae was always strongly supported [28,63]. Melanoplinae is the third largest subfamily of Acrididae, comprising 146 genera and 1196 species distributed throughout Eurasia and the Americas [19,57]. The origin of and the relationship within this subfamily were explored in a series of molecular studies [64,65,66,67,68,69]. As a morphologically well-characterized subfamily, the main diagnostic morphological characters of Melanoplinae are the smooth dorso-median carina of hind femora, the conical prosternal process with a sharply pointed apex, male 10th abdominal tergite with distinct furculae [16], the thick pallium, the species-specific male cerci [28], the male genitalia without sigmoid flexure but having a constricted point of articulation between the basal and apical penis valves [70], bridge-shaped, bridge broad, and undivided epiphallus, roughly trigonal ancorae and not detached from the bridge, elongated posterior projections of the epiphallus [42], and highly variable female genital complex [71].

Although *Alulacris* was considered most similar to the genus *Circocephalus* in the original reference [15], it is impossible to place the phylogenetic position of the genus *Alulacris* using the genus *Circocephalus* as a reference because the phylogenetic position of *Circocephalus* is also ambiguous. The genus *Circocephalus* was established by C. Willemse in 1928 and placed in the subfamily Catantopinae because of the presence of the prosternal process [72]. There is no further research on this genus except the description of two additional species [73,74], and its phylogenetic position was naturally classified by Otte to the subfamily Catantopinae with an uncertain tribe [57]. However, it was placed in the subfamily Coptacrinae by Bhowmik [75,76], making its position even more ambiguous.

**Figure 5:**
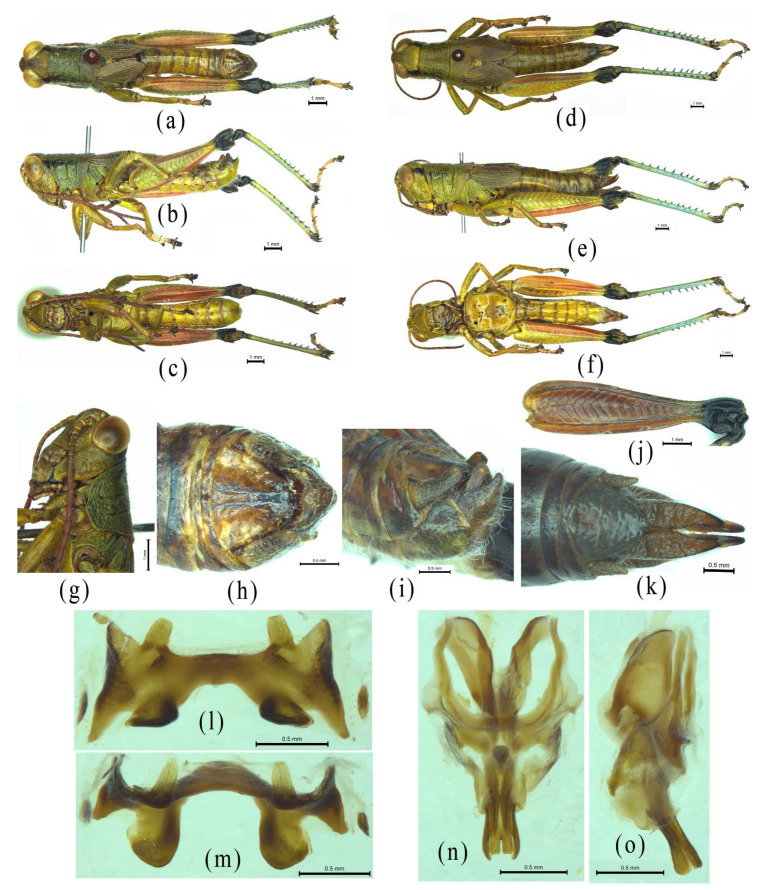
Morphological characters of *Alulacris* *shilinensis*. (**a**–**c**) Male habitus. (**d**–**f**) Female habitus. (**g**) Head and pronotum of male showing the shape of the prosternal process. (**h**) Terminal abdomen of a male in dorsal view showing the furculum. (**i**) Terminal abdomen of a male in lateral view showing the shape of cercus. (**j**) Hind femur of a male in lateral view showing the absence of denticles at the upper median keels. (**k**) Terminal abdomen of a female in dorsal view. (**l**,**m**) Epiphallus in dorsal and anterodorsal view. (**n**,**o**) Phallic complex in dorsal and lateral view.

**Figure 6:**
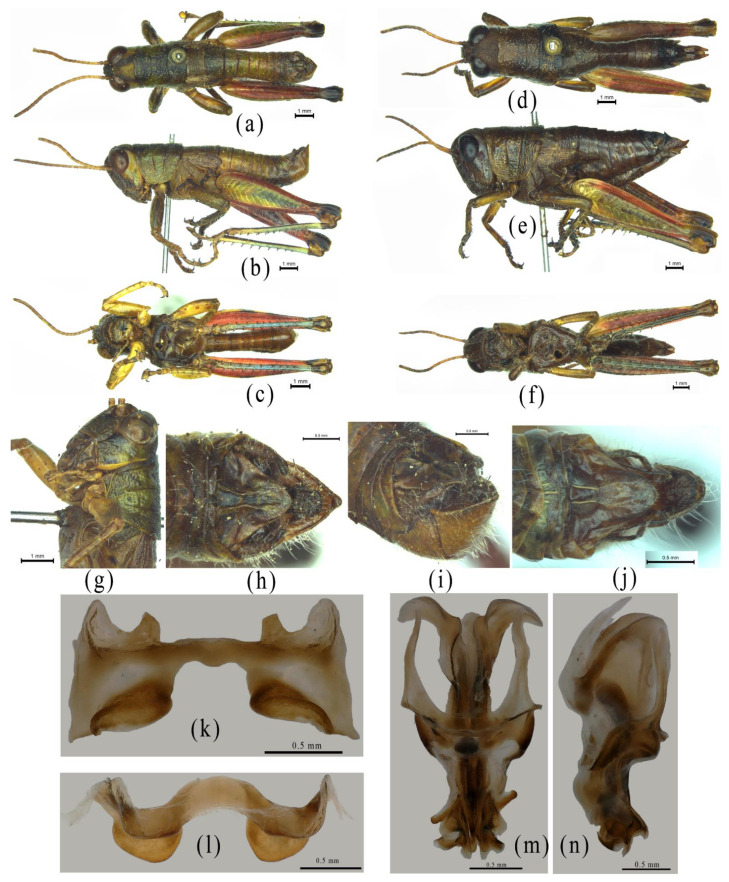
Morphological characters of *Yunnanacris* *yunnaneus*. (**a**–**c**) Male habitus. (**d**–**f**) Female habitus. (**g**) Head and pronotum of a male showing the shape of the prosternal process. (**h**) Terminal abdomen of a male in dorsal view showing the furculum. (**i**) Terminal abdomen of aale in lateral view, showing the shape of the cercus. (**j**) Terminal abdomen of a female in dorsal view. (**k**,**l**) Epiphallus in dorsal and anterodorsal view. (**m**,**n**) Phallic complex in dorsal and lateral view.

Despite the similarity of *Alulacris* to *Circocephalus* clearly stated by the original author [15], it was unambiguously considered as a member of the subfamily *Podisminaesensu* Li & Xia (2006) [16]. In this study, *Alulacris shilinensis* always falls into the clade of Melanoplinae and has closest the relationship with the genus *Yunnanacris* in all phylogenetic trees (Figure 1, Figure 2, Figure 3 and Figure 4). Morphologically, *Alulacris shilinensis* also highly matches the characteristics of Melanoplinae and is extremely similar to the genus *Yunnanacris* in the external appearance (Figure 5 and Figure 6), the shape of prosternal process (Figure 5g and Figure 6g), the small semicircular furculae (Figure 5h and Figure 6h), the epiphallus with long posterior projections, large inner lophi, and roughly trigonal ancorae (Figure 5l,m and Figure 6k,l), but differs from the latter in the size of the tegmina (Figure 5a,b,d,e vs. Figure 6a,b,d,e) and the shape of the valves of the cingulum and the apical valves of the penis (Figure 5n–o vs. Figure 6m–n). Therefore, the genus *Alulacris* is transferred here from Catantopinae *incertae sedis* to the nominal subtribe Podismina of the tribe Podismini *sensu* Ito (2015) [77] of the subfamily Melanoplinae. Moreover, it is possible that *Alulacris* may be a synonym of *Yunnanacris*, but more molecular data on the species of the tribe Podismini are needed to clarify the taxonomic status and composition of *Alulacris*, *Yunnanacris,* and related genera.

## Figures and Tables

**Table 1:** The best-fitting models used for the phylogenetic analyses.

Model Selection	Best Model	Partition Names
AICC	GTR + I + G	*ATP6*_pos1, *ATP6*_pos2, *ATP6*_pos3
		*ATP8*_pos1, *ATP8*_pos2
		*COX1*_pos1, *COX1*_pos2, *COX1*_pos3
		*COX2*_pos1, *COX2*_pos2, *COX2*_pos3
		*COX3*_pos2, *COX3*_pos3
		*CYTB*_pos1, *CYTB*_pos2, *CYTB*_pos3
		*ND1*_pos2, *ND1*_pos3
		*ND2*_pos1, *ND2*_pos2, *ND2*_pos3
		*ND3*_pos1, *ND3*_pos2
		*ND4*_pos1, *ND4*_pos2, *ND4*_pos3
		*ND4L*_pos3
		*ND5*_pos1, *ND5*_pos2, *ND5*_pos3
		*ND6*_pos3
	GTR + G	*COX3*_pos1
		*ND1*_pos1
		*ND3*_pos3
		*ND4L*_pos2
		*ND6*_pos1, *ND6*_pos2
		*RrnL*, *rrnS*
	HKY+I+G	*ATP8*_pos3
		*COX3*_pos2
		*ND4L*_pos1

**Table 2:** Nucleotide composition of the mitogenome of *Alulacris shilinensis*.

Feature	Length(bp)	A%	C%	G%	T%	A + T%	AT-skew	GC-skew
Whole genome	16,950	41.2	14.6	11.3	32.9	74.1	0.1120	−0.1274
Protein-coding genes	11,145	31.8	12.9	12.9	42.4	74.2	−0.1429	0.0000
First codon position	3715	32.0	12.5	20.3	35.5	67.5	−0.0519	0.2378
Second codon position	3715	19.4	20.5	14.3	45.8	65.2	−0.4049	−0.1782
Third codon position	3715	43.9	5.6	4.1	46.4	90.3	−0.0277	−0.1546
Protein-coding genes-J	6867	35.9	15.2	12.3	29.1	65.0	0.1046	−0.1055
First codon position	2289	35.0	15.2	20.8	29.1	64.1	0.0920	0.1556
Second codon position	2289	19.8	22.2	13.7	44.3	64.1	−0.3822	−0.2368
Third codon position	2289	25.8	8.3	2.4	36.5	62.3	−0.1717	−0.5514
Protein-coding genes-N	4278	25.2	9.1	14.0	51.8	77.0	−0.3455	0.2121
First codon position	1426	27.1	8.3	19.6	45.0	72.1	−0.2483	0.4050
Second codon position	1426	18.7	17.7	15.4	48.2	66.9	−0.4410	−0.0695
Third codon position	1426	29.7	2.3	6.9	62.2	91.9	−0.3536	0.5000
*ATP6*	678	37.9	15.3	9.6	37.2	75.1	0.0093	−0.2289
*ATP8*	162	43.8	12.3	6.2	37.7	81.5	0.0748	−0.3297
*COX1*	1540	33.6	15.5	15.5	35.4	69.0	−0.0261	0.0000
*COX2*	684	36.5	16.1	13.6	33.8	70.3	0.0384	−0.0842
*COX3*	792	32.7	17.7	14.5	35.1	52.8	−0.3295	−0.0994
*CYTB*	1137	34.3	14.7	12.5	38.5	72.8	−0.0577	−0.0809
*ND1*	942	50.1	13.3	9.8	26.9	77.0	0.3013	−0.1515
*ND2*	1018	37.8	15.6	36.5	25.6	63.4	0.1924	0.4012
*ND3*	353	35.7	14.2	12.2	38.0	73.7	−0.0312	−0.0758
*ND4*	1335	52.5	14.5	8.8	24.3	76.8	0.3672	−0.2446
*ND4L*	294	57.8	14.3	7.5	20.4	78.2	0.4783	−0.3119
*ND5*	1719	51.0	13.8	9.2	25.9	76.9	0.3264	−0.2000
*ND6*	522	42.0	10.7	6.9	40.4	82.4	0.0194	−0.2159
*rrnL*	1377	44.4	15.0	8.9	31.7	76.1	0.1669	−0.2552
*rrnS*	793	39.8	16.6	10.5	33.0	72.8	0.0934	−0.2251
D-loop	2121	35.6	13.5	14.1	36.9	72.5	−0.0179	0.0217

**Table 3:** Annotation of the complete mitogenome of *Alulacris shilinensis*.

Name	Type	Minimum	Maximum	Length	Direction	START	END	Interval
*trnI*	tRNA	1	66	66	+			4
*trnQ*	tRNA	71	139	69	−			1
*trnM*	tRNA	141	209	69	+			0
*ND2*	CDS	210	1228	1019	+	ATG	TA	2
*trnW*	tRNA	1231	1298	68	+			−8
*trnC*	tRNA	1291	1355	65	−			8
*trnY*	tRNA	1364	1429	66	−			−8
*COX1*	CDS	1422	2961	1540	+	ATC	T	0
*trnL2*	tRNA	2962	3027	66	+			4
*COX2*	CDS	3032	3715	684	+	ATG	TAA	−2
*trnD*	tRNA	3714	3778	65	+			2
*trnK*	tRNA	3781	3851	71	+			14
*ATP8*	CDS	3866	4027	162	+	ATC	TAA	−7
*ATP6*	CDS	4021	4698	678	+	ATG	TAA	4
*COX3*	CDS	4703	5494	792	+	ATG	TAA	2
*trnG*	tRNA	5497	5563	67	+			0
*ND3*	CDS	5564	5917	354	+	ATT	TAA	−1
*trnA*	tRNA	5917	5981	65	+			2
*trnR*	tRNA	5984	6048	65	+			3
*trnN*	tRNA	6052	6120	69	+			0
*trnS1*	tRNA	6121	6187	67	+			0
*trnE*	tRNA	6188	6253	66	+			0
*trnF*	tRNA	6254	6319	66	−			1
*ND5*	CDS	6321	8039	1719	−	ATT	TAA	15
*trnH*	tRNA	8055	8119	65	−			4
*ND4*	CDS	8124	9458	1335	−	ATG	TAA	−7
*ND4L*	CDS	9452	9745	294	−	ATG	TAA	2
*trnT*	tRNA	9748	9816	69	+			0
*trnP*	tRNA	9817	9880	64	−			2
*ND6*	CDS	9883	10,404	522	+	ATG	TAA	6
*CYTB*	CDS	10,411	11,550	1140	+	ATG	TAA	1
*trnS2*	tRNA	11,552	11,621	70	+			16
*ND1*	CDS	11,638	12,582	945	−	ATA	TAG	3
*trnL1*	tRNA	12,586	12,651	66	−			−51
*rrnL*	rRNA	12,601	13,977	1377	−			−14
*trnV*	tRNA	13,964	14,034	71	−			2
*rrnS*	rRNA	14,037	14,829	793	−			0
D-loop	unsure	14,830	16,950	2121	+			

**Table 4:** Codon usage of the PCGs of *Alulacrisshilinensis*.

Amino Acid	Codon	No.	RSCU (%)	Amino Acid	Codon	No.	RSCU (%)
Phe	UUU	297	1.73	Tyr	UAU	143	1.73
	UUC	47	0.27		UAC	22	0.27
Leu	UUA	369	4.13	End	UAA	0	0
	UUG	30	0.34		UAG	0	0
Leu	CUU	74	0.83	His	CAU	55	1.53
	CUC	10	0.11		CAC	17	0.47
	CUA	49	0.55	Gln	CAA	60	1.85
	CUG	4	0.04		CAG	5	0.15
Ile	AUU	334	1.8	Asn	AAU	144	1.71
	AUC	37	0.2		AAC	24	0.29
Met	AUA	227	1.79	Lys	AAA	65	1.41
	AUG	27	0.21		AAG	27	0.59
Val	GUU	104	2.12	Asp	GAU	68	1.74
	GUC	3	0.06		GAC	10	0.26
	GUA	84	1.71	Glu	GAA	68	1.68
	GUG	5	0.1		GAG	13	0.32
Ser	UCU	123	2.71	Cys	UGU	41	1.91
	UCC	5	0.11		UGC	2	0.09
	UCA	126	2.78	Trp	UGA	89	1.78
	UCG	1	0.02		UGG	11	0.22
Pro	CCU	67	1.99	Arg	CGU	17	1.19
	CCC	5	0.15		CGC	0	0
	CCA	60	1.78		CGA	38	2.67
	CCG	3	0.09		CGG	2	0.14
Thr	ACU	63	1.3	Ser	AGU	30	0.66
	ACC	11	0.23		AGC	3	0.07
	ACA	117	2.41		AGA	75	1.65
	ACG	3	0.06		AGG	0	0
Ala	GCU	76	1.72	Gly	GGU	86	1.54
	GCC	8	0.18		GGC	4	0.07
	GCA	89	2.01		GGA	115	2.05
	GCG	4	0.09		GGG	19	0.34

## Data Availability

The mitogenome sequence of *Alulacrisshilinensis* is deposited in GenBank under accession number MW810985.

## Supplementary Materials

The following are available online at https://www.mdpi.com/article/10.3390/insects12100918/s1, Figure S1. Secondary structures of 22 tRNAs of the nine newly sequenced mitogenomes. Figure S2. Phylogenetic tree reconstructed from sequences of the two rRNAs using maximum likelihood. The asterisk indicates the species *Alulacris* *shilinensis*. Figure S3. Phylogenetic tree reconstructed from sequences of the two rRNAs using Bayesian inference. The asterisk indicates the species *Alulacris* *shilinensis*. Table S1. Accession numbers and references of the mitogenomes of the species sampled in this study.
