# Supplementary material for: Phylogenetic Position of the Genus Alulacris (Orthoptera: Acrididae: Melanoplinae: Podismini) Revealed by Complete Mitogenome Evidence
Source: Insects. 2021 Oct 8;12(10):918. doi: 10.3390/insects12100918 (PMC8539312; doi:10.3390/insects12100918)
Supplement: Supplementary file 1 [file insects-12-00918-s001.zip › Figure S1-S3.pdf]

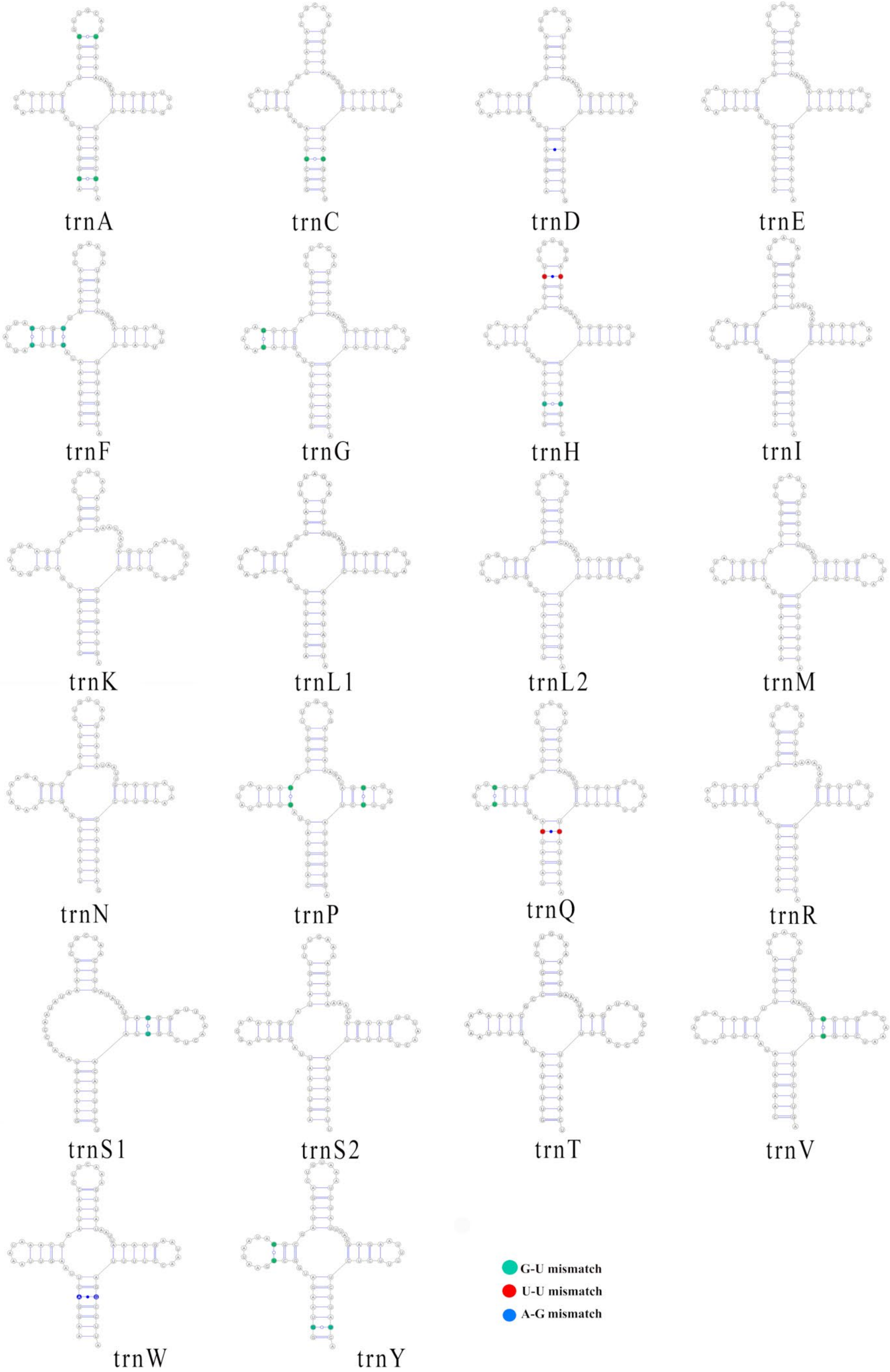

Figure S1. Secondary structures of 22 tRNAs of the nine newly sequences mitogenomes.

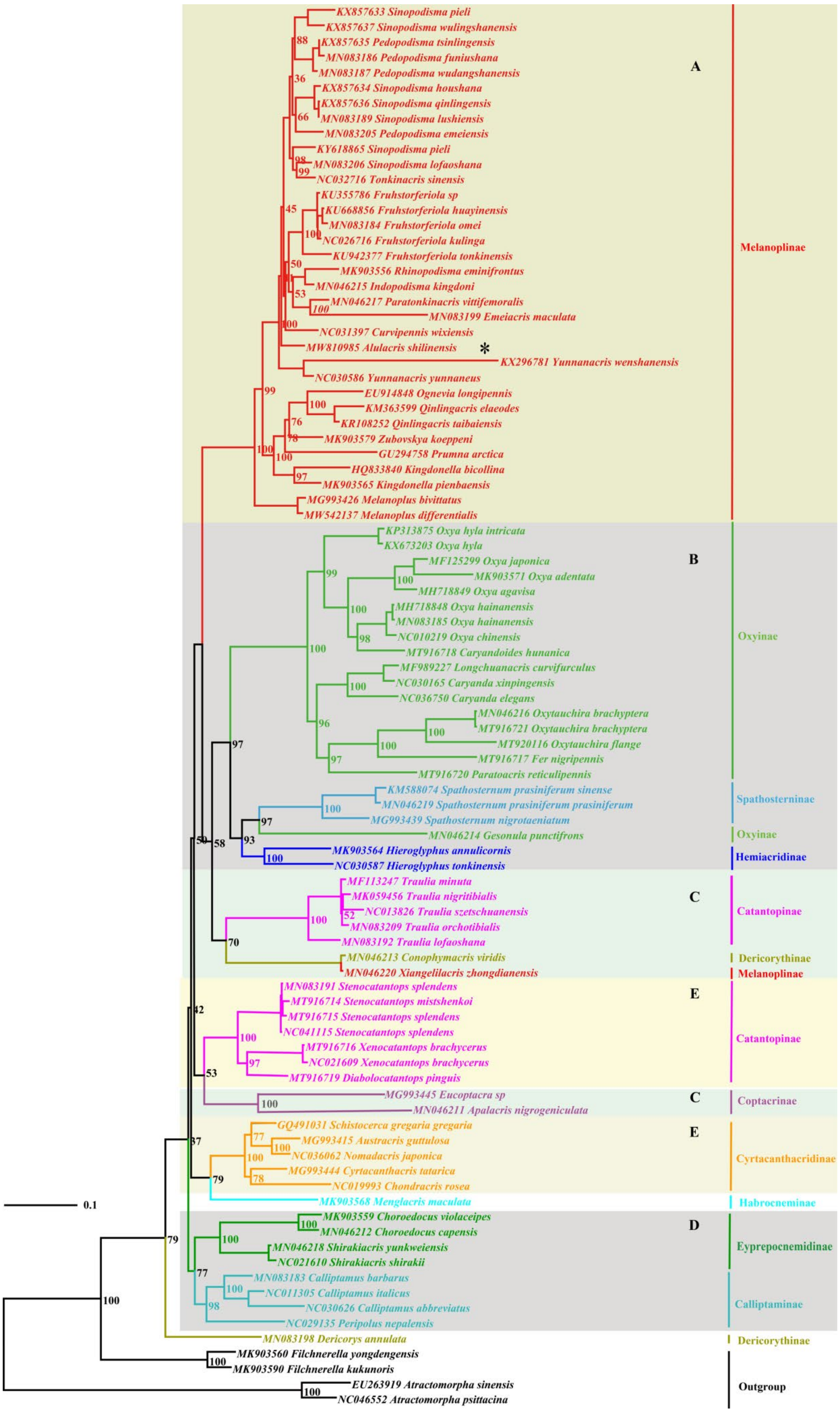

Figure S2. Phylogenetic tree reconstructed from sequences of the 2 rRNAs using maximum likelihood. The asterisk indicates the species *Alulacris shilinensis*.

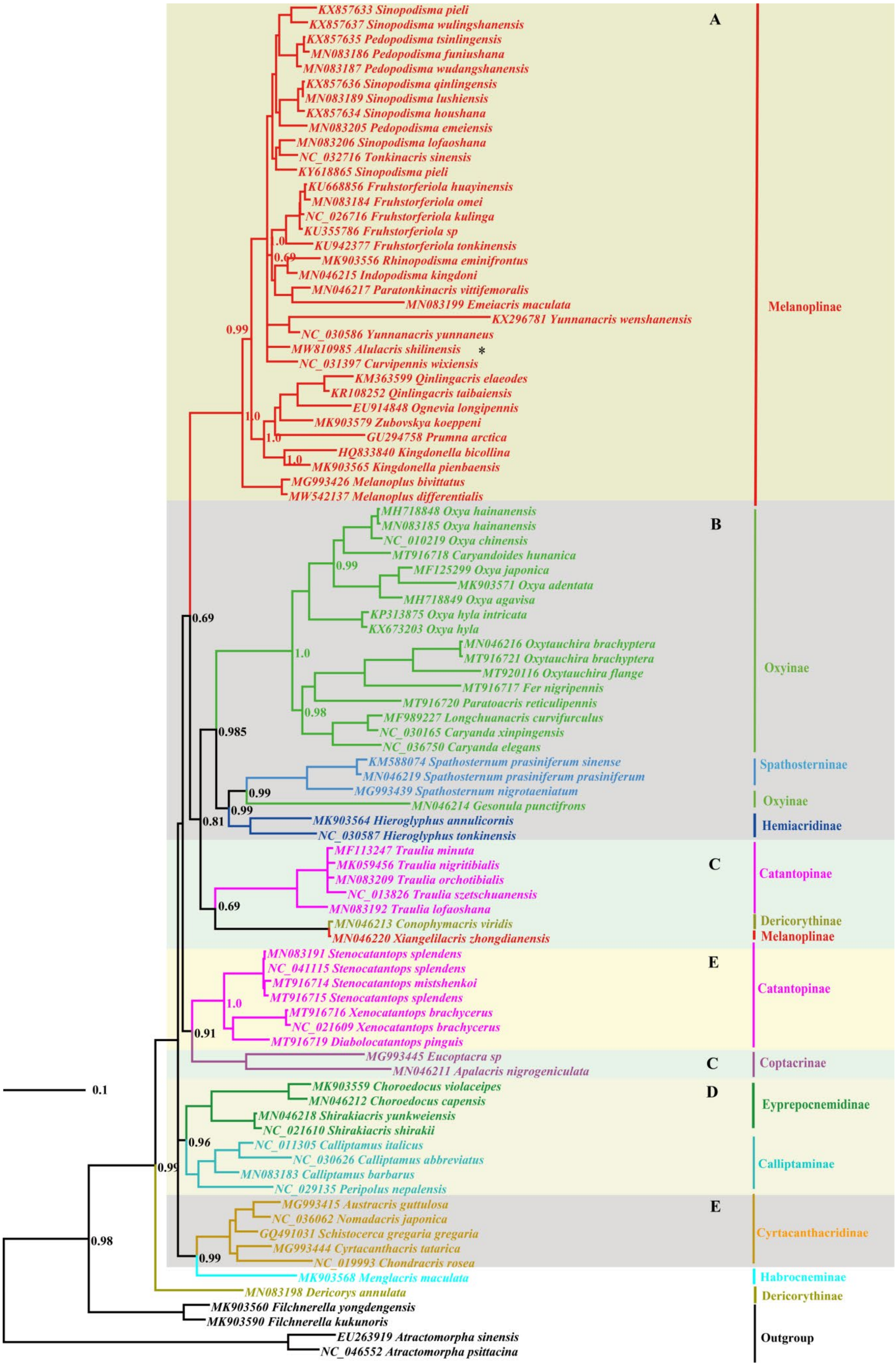

Figure S3. Phylogenetic tree reconstructed from sequences of the 2 rRNAs using Bayesian inference. The asterisk indicates the species *Alulacris shilinensis*.
