# Supplementary material for: Phylogenetic Position of the Genus Alulacris (Orthoptera: Acrididae: Melanoplinae: Podismini) Revealed by Complete Mitogenome Evidence
Source: Insects. 2021 Oct 8;12(10):918. doi: 10.3390/insects12100918 (PMC8539312; doi:10.3390/insects12100918)
Supplement: Supplementary file 1 [file insects-12-00918-s001.zip › Table S1.pdf]

Table S1. Accession numbers and references of the mitogenomes of the species sampled in this study.

Species	Accession number	Reference
**Caelifera, Acridoidea, Acrididae**		
**Calliptaminae**		
Calliptamus abbreviatus	NC_030626	Han *et al.*, 2016
Calliptamus barbarus	MN083183	Chang *et al.* 2020a
Calliptamus italicus	NC_011305	Fenn *et al.*, 2008
Peripolus nepalensis	NC_029135	Zhi *et al.*, 2016a
**Catantopinae**		
Diabolocatantops pinguis	MT916719	Zeng *et al.*, 2021
Stenocatantops mistshenkoi	MT916714	Zeng *et al.*, 2021
Stenocatantops splendens	MT916715	Zeng *et al.*, 2021
Stenocatantops splendens	NC_041115	Li *et al.* 2019
Stenocatantops splendens	MN083191	Chang *et al.* 2020a
Traulia lofaoshana	NC_046551	Chang *et al.* 2020a
Traulia minuta	NC_036063	Direct submission
Traulia nigritibialis	NC_041114	Li *et al.* 2019
Traulia orchotibialis	NC_046565	Chang *et al.* 2020a
Traulia szetschuanensis	NC_013826	Direct submission
Xenocatantops brachycerus	MT916716	Zeng *et al.*, 2021
Xenocatantops brachycerus	NC_021609	Yang, J *et al.* 2016
**Coptacrinae**		
Apalacris nigrogeniculata	NC_046527 (=MN046211)	Chang *et al.* 2020a
Eucoptacra sp	MG993445	Song *et al.* 2018
**Cyrtacanthacridinae**		
Austracris guttulosa	MG993415	Direct submission
Chondracris rosea	NC_019993	Direct submission
Cyrtacanthacris tatarica	MG993444	Direct submission
Patanga japonica	NC_036062	Direct submission
Schistocerca gregaria gregaria	NC_013240	Erler *et al.*, 2010
**Eyprepocnemidinae**		
Choroedocus capensis	MN046212	Chang *et al.* 2020a
Choroedocus violaceipes	MK903559	Chang *et al.* 2020b
Shirakiacris shirakii	NC_021610	Direct submission
Shirakiacris yunkweiensis	MN046218	Chang *et al.* 2020a
**Habrocneminae**		
Menglacris maculata	MK903568	Chang *et al.* 2020b
**Hemiacridinae**		
Hieroglyphus annulicornis	MK903564	Chang *et al.* 2020b
Hieroglyphus tonkinensis	NC_030587	Direct submission
Leptacris sp.	MG993429	Song *et al.* 2018
**Melanoplinae**		
Alulacris shilinensis	MW810985	This study
Rhinopodisma eminifrontus	MK903556	Chang *et al.* 2020b

Curvipennis wixiensis	NC_031397	Chen and Xu, 2017
Emeiacris maculata	MN083199	Chang *et al*. 2020a
Fruhstorferiola huayinensis	KU668856	Liu and Qiu, 2016
Fruhstorferiola kulinga	NC_026716	Yang, R *et al*., 2016
Fruhstorferiola omei	MN083184	Chang *et al*. 2020a
Fruhstorferiola sp.	KU355786	Direct submission
Fruhstorferiola tonkinensis	KU942377	Zhang and Lin 2016
Indopodisma kingdoni	MN046215	Chang *et al*. 2020a
Kingdonella bicollina	NC_023920	Zhi et al., 2016b
Kingdonella pienbaensis	MK903565	Chang *et al*. 2020b
Melanoplus bivittatus	MG993426	Song *et al*., 2018
Melanoplus differentialis	MW542137	Direct submission
Ognevia longipennis	NC_013701	Direct submission
Paratonkinacris vittfemoralis	MN046217	Chang *et al*. 2020a
Pedopodisma funiushana	MN083186	Chang *et al*. 2020a
Pedopodisma wudangshanensis	MN083187	Chang *et al*. 2020a
Pedopodisma emeiensis	MN083205	Chang *et al*. 2020a
Pedopodisma tsinlingensis	KX857635	Direct submission
Prumna arctica	NC_013835	Sun et al., 2010
Qinlingacris elaeodes	KM363599	Li *et al*., 2016
Qinlingacris taibaiensis	NC_027187	Direct submission
Sinopodisma houshana	KX857634	Qiu *et al*., 2020
Sinopodisma lofaoshana	MN083206	Chang *et al*. 2020a
Sinopodisma lushiensis	MN083189	Chang *et al*. 2020a
Sinopodisma pieli	KX857633	Qiu *et al*., 2020
Sinopodisma pieli	KY618865	Liu *et al*., 2017
Sinopodisma qinlingensis	KX857636	Qiu *et al*., 2020
Sinopodisma wulingshana	KX857637	Qiu *et al*., 2020
Tonkinacris sinensis	NC_032716	
Xiangelilacris zhongdianensis	MN046220	Chang *et al*. 2020a
Yunnanacris wenshanensis	KX296781	Direct submission
Yunnanacris yunnaneus	NC_030586	Hu *et al*., 2016
Zubovskia koeppeni	MK903579	Chang *et al*. 2020b
**Oxyinae**		
Caryanda neoelegans	NC_036750	Yuan *et al*. 2019
Caryanda xinpingensis	NC_030165	Hu *et al*. 2017
Caryandoides hunanica	MT916718	Zeng *et al*., 2021
Fer nigripennis	MT916717	Zeng *et al*., 2021
Gesonula punctifrons	MN046214	Chang *et al*. 2020a
Longchuanacris curvifurculus	NC_036994	Hu *et al*. 2018
Oxya adentata	MK903571	Chang *et al*. 2020b
Oxya agavisa	NC_045883 (=MH718849)	Li *et al*., 2020b
Oxya chinensis	NC_010219	Zhang and Huang 2008
Oxya hainanensis	NC_045928 (=MH718848)	Li *et al*., 2020b

Oxya hainanensis	MN083185	Chang *et al.* 2020a
Oxya hyla	NC_032076 (=KX673203)	Song *et al.*, 2016
Oxya hyla intricata	KP313875	Dong *et al.* 2016
Oxya japonica	NC_043773 (=MF125299)	Li *et al.*, 2020b
Oxytauchira brachyptera	MN046216	Chang *et al.* 2020a
Oxytauchira brachyptera	MT916721	Zeng *et al.*, 2021
Oxytauchira flange	MT920116	Zeng *et al.*, 2021
Paratoacris reticulipennis	MT916720	Zeng *et al.*, 2021
Pseudoxya diminuta	NC_025765	Tang *et al.* 2014
**Spathosterninae**		
Spathosternum nigrotaeniatum	MG993439	Song *et al.* 2018
Spathosternum prasiniferum Prasiniferum	NC_046532	Chang *et al.* 2020a
Spathosternum prasiniferum Sinense	KM588074	Zhou and Huang 2016
**Dericorythidae, Conophyminae**		
Conophymacris viridis	MN046213	Chang *et al.* 2020a
**Dericorythinae**		
Dericorys annulata	MN083198	Chang *et al.* 2020a
**Pamphagidae**		
Filchnerella yongdengensis	MK903560	Chang *et al.* 2020b
Filchnerella kukunoris	MK903590	Chang *et al.* 2020b
**Pyrgomorphoidea, Pyrgomorphidae, Pyrgomorphinae**		
Atractomorpha sinensis	EU263919	Ding *et al.*, 2007
Atractomorpha psittacina	NC_046552	Chang *et al.* 2020a

References

Chang, H., Nie, Y., Zhang, N., Zhang, X., Sun, H., Mao, Y., Qiu, ZY, and Huang, Y. MtOrt: An empirical mitochondrial amino acid substitution model for evolutionary studies of Orthoptera insects. *BMC Evolutionary Biology* 2020a, **20**, 57.

Chang, H., Qiu, Z., Yuan, H., Wang, X., Li, X., Sun, H., Guo, X., Lu, Y., Feng, X., Majid, M., Huang, Y. Evolutionary rates of and selective constraints on the mitochondrial genomes of Orthoptera insects with different wing types. *Molecular Phylogenetics and Evolution* 2020b,. 145, 106734.

Chen Z N , Xu S Q . The complete mitochondrial DNA genome sequence of a terrestrial grasshopper, *Curvipennis wixiensis* (Acrididae: Podismini). Conservation Genetics Resources, 2017, 9(1): 115–118.

Ding F., Shi H., Huang Y. Complete Mitochondrial Genome and Secondary Structures of lrRNA and srRNA of *Atractomorpha sinensis* (Orthoptera, Pyrgomorphidae). Zoological Research 2007, 28(6), 580–588.

Dong, J.J., Guan, D.L, and Xu, S.Q. Complete mitogenome of the semi-aquatic grasshopper *Oxya intricata* (Stal.) (Insecta: Orthoptera: Catantopidae). *Mitochondrial DNA, Part A* 2016, **27(5)**, 3233–3234.

Erler, S., Ferenz, H.-J., Moritz, R.F.A., Kaatz, H.-H.. Analysis of the mitochondrial genome of *Schistocerca gregaria gregaria* (Orthoptera: Acrididae). Biological Journal of the Linnean Society 2010, 99(2), 296–305.

Fenn, J.D.; Song, H.; Cameron, S.L.; Whiting, M.F. A preliminary mitochondrial genome phylogeny of Orthoptera (Insecta) and approaches to maximizing phylogenetic signal found within mitochondrial genome data. Molecular Phylogenetics and Evolution 2008, 49, 59–68.

Han H, Wang N, Xu L, Gao, S.; Liu, A. The complete mitochondrial genome of *Calliptamus abbreviatus* Ikovnnikov (Orthoptera: acridoidea). Mitochondrial DNA Part B, 2016, 1(1), 770–771.

Hu Z., Guan D.-L., Mao, B.-Y. Characterization of the complete mitochondrial genome of the Yunnan endemic grasshopper *Yunnanacris yunnaneus* (Insecta: Orthoptera: Acrididae). Conservation Genet. Resour. 2016, 8, 267–270.

Hu, Z., Guan, D.L, and Mao, B.Y. Description of a new species, *Caryanda* Stal, 1878 (Acrididae, Orthoptera) from China. *Oriental insects* 2017, **51(2)**, 124–134.

Hu, Z., Han, Y.P., Guan, D.L, and Mao, B.Y. Characterization of the complete mitochondrial genome of the Yunnan endemic grasshopper *Longchuanacris curvifurculus* (Insecta: Orthoptera: Catantopidae). *Mitochondrial DNA, Part B* 2018, **3(2)**, 670–671.

Li R, Jiang G F, Liang A P, Zhong, X.T., Liu, Y. Characterization of the mitochondrial genome of the montane grasshopper, *Qinlingacris elaeodes* (Orthoptera: Catantopidae). Mitochondrial DNA Part A, 2016, 27(3): 1765–1766.

Li, R.; Shu, X.; Li, X.; Meng, L.; Li B. Comparative mitogenome analysis of three species and monophyletic inference of Catantopinae (Orthoptera: Acridoidea). Genomics 2019, 111, 1728–1735.

Li, R.; Shu, X.; Deng, W.; Meng, L.; Li B. Complete mitochondrial genome of *Atractomorpha sagittaris* (Orthoptera: Pyrgomorphidae) and its phylogenetic analysis for Acrididea. Biologia 2020a, 75(10), 1571–1583.

Li, R.; Wang Y.; Shu, X.; Meng, L.; Li B. Complete mitochondrial genomes of three *Oxya* grasshoppers (Orthoptera) and their implications for phylogenetic reconstruction. Genomics 2020b, 112, 289–296.

Liu F, Qiu Z. The complete mitochondrial genome of *Fruhstorferiola huayinensis* (Orthoptera: Catantopidae). Mitochondrial DNA Part B, 2016, 1(1): 273-274.

Qiu, Z.; Chang, H.; Yuan, H.; Huang, Y.; Lu, H.; Li, X.; Gou, X. Comparative mitochondrial genomes of four species of *Sinopodisma* and phylogenetic implications (Orthoptera, Melanoplinae). ZooKeys 2020, 969, 23–42.

Song, H., Mariño-Pérez, R., Woller, D.A, and Cigliano, M.M. Evolution, Diversification, and Biogeography of Grasshoppers (Orthoptera: Acrididae). *Insect Systematics and Diversity* 2018, **2(4)**, 3; 1–25.

Song N, Li H, Song F, Cai W. Molecular phylogeny of Polyneoptera (Insecta) inferred from expanded mitogenomic data. Sci Rep. 2016, 6(1), 36175.

Sun H , Zheng Z , Huang Y . Sequence and phylogenetic analysis of complete mitochondrial DNA genomes of two grasshopper species *Gomphocerus rufus* (Linnaeus, 1758) and *Primnoa arctica* (Zhang and Jin, 1985) (Orthoptera: Acridoidea). Mitochondrial DNA, 2010, 21(3-4), 115–131.

Tang, M., Tan, M.H., Meng, G.L., Yang, S.Z., Su, X., Liu, S.L., Song, W.H., Li, Y.Y., Wu, Q., Zhang, A.B, and Zhou, X. Multiplex sequencing of pooled mitochondrial genomes-a crucial step toward biodiversity analysis using mito-metagenomics. *Nucleic Acids Research* 2014, **42(22)**, e166.

Yang, J., Liu, Y, and Liu, N. The complete mitochondrial genome of the *Xenocatantops brachycerus* (Orthoptera: Catantopidae). *Mitochondrial DNA, Part A* 2016, **27(4)**, 2844–2845.

Yang R., Guan D.-L., Xu S-Q. Complete mitochondrial genome of the Chinese endemic grasshopper *Fruhstorferiola kulinga* (Orthoptera: Acrididae: Podismini). Mitochondrial DNA Part A 2016, 27(5), 3240-3241.

Yuan, H., Qiu, Z.Y., Yang, C, and Huang, Y. The complete mitochondrial genome sequence of *Caryanda elegans* (Orthoptera: Acrididae). *Mitochondrial DNA, Part B* 2019, **4(1)**, 1580–1581.

Zeng X, Xu HY, Gu JX, Mao BY, Chen ZL, Huang Y, Huang JH. Phylogenetic position of the genera Caryandoides, Paratoacris, Fer and Longchuanacris (Orthoptera : Acrididae) revealed by complete mitogenome sequences. Invertebrate Systematics, 2021, https://doi.org/10.1071/IS20077.

Zhang, C.Y, and Huang, Y. Complete mitochondrial genome of *Oxya chinensis* (Orthoptera, Acridoidea). *Acta Biochimica et Biophysica Sinica* 2008, **40(1)**, 7–18.

Zhang X., Lin L. The complete mitochondrial genome of *Fruhstorferiola tonkinensis* (Orthoptera: Catantopidae), Mitochondrial DNA Part B, 2016, 1(1), 434–435.

Zhang X., Li X., Liu F., Yuan H., Huang Y. The complete mitochondrial genome of *Tonkinacris sinensis* (Orthoptera: Acrididae): A tRNA-like sequence and its implications for phylogeny. Biochemical Systematics and Ecology 2017, 70,147–154.

Zhi, Y., Zhang, N., Lu, X., Yin, H., Zhang D. The complete mitochondrial genome of *Peripolus nepalensis* Uvarov, 1942 (Orthoptera: Acridoidea: Catantopidae). Mitochondrial DNA, 2016a, 27(1), 26–27.

Zhi Y , Liu B , Han G , Yin H., Zhang D.C. The complete mitochondrial genome of *Kingdonella bicollina* (Orthoptera: Acridoidea: Catantopidae). Mitochondrial DNA Part A 2016b, 27(1): 391–392.

Zhou, F., Huang, Y. The complete mitochondrial genome of *Spathosternum prasiniferum sinense* Uvarov, 1931 (Orthoptera: Acridoidea: Acrididae). Mitochondrial DNA Part A, 2016, 27(3): 1932-1933.
